# Co-circulation of coxsackieviruses A-6, A-10, and A-16 causes hand, foot, and mouth disease in Guangzhou city, China

**DOI:** 10.1186/s12879-020-04992-x

**Published:** 2020-04-07

**Authors:** Jia Xie, Xiao-Han Yang, Si-Qi Hu, Wen-Li Zhan, Chang-Bin Zhang, Hong Liu, Hong-Yu Zhao, Hui-Ying Chai, Ke-Yi Chen, Qian-Yi Du, Pan Liu, Ai-Hua Yin, Ming-Yong Luo

**Affiliations:** 1 Medical Genetic Centre, Guangdong Women and Children’s Hospital, Guangzhou Medical University, Guangzhou, 511442 People’s Republic of China; 2 Medical Genetic Centre, Guangdong Women and Children Hospital, Guangzhou, 511442 People’s Republic of China; 3 Department of Pediatrics, Guangdong Women and Children Hospital, Guangzhou, 511442 People’s Republic of China

**Keywords:** Enterovirus, Coxsackievirus, Co-circulation, Hand, foot, and mouth disease

## Abstract

**Background:**

Hand, foot, and mouth disease (HFMD) is a common infectious disease occurring in children under 5 years of age worldwide, and Enterovirus A71 (EV-A71) and Coxsackievirus A16 (CVA-16) are identified as the predominant pathogens. In recent years, Coxsackievirus A6 (CVA-6) and Coxsackievirus A10 (CVA-10) have played more and more important role in a series of HFMD outbreaks. This study aimed to understand the epidemic characteristics associated with HFMD outbreak in Guangzhou, 2018.

**Methods:**

The clinical and laboratory data of 1220 enterovirus-associated HFMD patients in 2018 were analysed in this study. Molecular diagnostic methods were performed to identify its serotypes. Phylogenetic analyses were depicted based on the complete VP1 gene.

**Results:**

There were 21 enterovirus serotypes detected in Guangzhou in 2018. Three serotypes of enterovirus, CVA-6 (364/1220, 29.8%), CVA-10 (305/1220, 25.0%), and CVA-16 (397/1220, 32.5%), were identified as the causative pathogens and accounted for 87.3% among all 1220 HFMD patients. In different seasons, CVA-6 was the predominant pathogen of HFMD during autumn, and CVA-10 as well as CVA-16 were more prevalent in summer. Patients infected by CVA-6, CVA-10 or CVA-16 showed similar clinical features and laboratory characteristics, and the ratios of severe HFMD were 5.8, 5.9, and 1.5% in the three serotypes. Phylogenetic analyses of VP1 sequences showed that the CVA-6, CVA-10, and CVA-16 sequences belonged to the sub-genogroup E2, genogroup E, and genogroup B1, respectively.

**Conclusions:**

CVA-6, CVA-10, and CVA-16 were the predominant and co-circulated serotypes in Guangzhou China, 2018, which should be the new target for prevention and control of HFMD. Our findings provide useful information for diagnosis, treatment, and prevention of HFMD.

## Background

Hand, foot, and mouth disease (HFMD), a common contagious disorder in children under 5 years of age, is characterised by a brief, generally mild, febrile illness with multiple oral ulcers, and eruption of vesiculo-papular rashes over the hands, feet, mouth, and buttocks [1–3]. Some cases can develop severe neurological and systemic complications, such as aseptic meningitis, acute flaccid paralysis, encephalitis, cardiorespiratory failure, and severe pulmonary oedema, and can even lead to death [4, 5]. HFMD is mainly caused by human enteroviruses (EVs) of the family *Picornaviridae*. Over 100 recognised EV serotypes exist, classified into four groups, namely, EV-A to EV-D [6]. Their genome comprises one open reading frame, which encodes four structural viral proteins (VP1, VP2, VP3, and VP4) and seven non-structural proteins (2A-2C and 3A-3D) [7]. VP1, the main region encoding neutralising epitopes, contains virulence determinants and also provides conclusive evidence regarding the phylogeny and genotype of these viruses [8].

Since the first HFMD case was described in 1959 [9], outbreaks or sporadic cases have frequently occurred globally [10–13]. In the recent decades, more and more outbreaks of HFMD had reported from Asia-Pacific region, including Vietnam, Malaysia, Thailand, Japan, India, and China [10, 13–17]. In China, HFMD was recognised as a C-class notifiable disease after an outbreak occurred in Fuyang in early 2008 [17, 18]. According to the Chinese national enhanced surveillance system, HFMD rates ranked first among all notifiable diseases and over 1.2/1000 in children are affected annually [3]. Enterovirus A71 (EV-A71) and coxsackievirus A16 (CVA-16), belonging to EV-A, have been identified as the predominant causative pathogens in the nationwide surveillance. However, other serotypes had gradually played an important role in the HFMD outbreaks. Since a Coxsackievirus A6 (CVA-6) associated HFMD outbroke in Finland in 2008, several outbreaks caused by CVA-6 were reported in Europe, North America, and Asia [12, 19–22]. Moreover, Coxsackievirus A10 (CVA-10) had also been identified as the responsible pathogen for a series of HFMD outbreaks [20, 23, 24]. Guangzhou city is one of the most serious cities in HFMD with high incidence, and the HFMD pathogen spectrum are changing according to previous studies [22, 25–27]. Therefore, the change of HFMD pathogen spectrum has become a big challenge for prevention and control of HFMD.

In this study, the clinical and epidemiological characteristics of HFMD were analysed in Guangzhou, 2018, and the evolutionary dynamics were also explicated based on entire VP1 sequences.

## Methods

### Case definitions and classification

The criteria for clinical HFMD diagnosis was consistent with Chinese guidelines for the diagnosis and treatment of HFMD [1]. Mild HFMD cases were defined as patients having oral ulcers and maculopapular or vesicular rashes on their hands, feet, and buttocks, with or without fever. Severe HFMD cases were defined as HFMD accompanied by at least one of the following neurological and cardiopulmonary complications: aseptic meningitis, encephalitis, encephalomyelitis, acute flaccid paralysis, autonomic nervous system dysregulation, pulmonary oedema, pulmonary haemorrhage, and cardiorespiratory failure.

### Patients and data collection

All HFMD patients with laboratory-confirmed EV infection at the Paediatric Department of Guangdong Women and Children Hospital between January 2018 and December were included in this study. Demographic data (sex and age range) and clinical manifestations (cough, diarrhea, vomiting, fever spike, neurologic complications, and skin lesions) were collected. Laboratory data, including hypersensitive C-reactive protein (hsCRP), white blood cell (WBC), neutrophils, lymphocyte, platelet (PLT), aspartate aminotransferase (AST), creatine kinase (CK), creatine kinase-MB (CK-MB), were also collected.

This study was approved by the ethics committee of the Guangdong Women and Children Hospital (ref.201901117). The requirement for written informed consent was waived as virological testing for patients who underwent regular medical examination at the hospital was a routine diagnostic procedure. All information collected from patients was delinked from individual patient identifiers.

### Statistical analysis

The data were analysed using SPSS 19.0 (Chicago, USA). The categorical variables are presented as frequencies and percentages, and quantitative data are reported as the median (interquartile range). Chi-square test or Fisher’s exact tests were used for categorical data, and the quantitative data were analysed using non-parametric rank sum test (Kruskal-Wallis test) [28, 29]. Two-sided *P* value < 0.05 was considered statistically significant.

### EV detection and serotype determination

A total of 1322 clinical samples, including rectal swabs or fecal sample (*n* = 1138), throat swabs (*n* = 85), vesicular fluid (*n* = 21), and cerebrospinal fluid (*n* = 78), were collected from positive patients (*n* = 1220). RNA was extracted using the QIAamp viral RNA mini kit (Qiagen, Germany) [22]. Firstly, samples were identified by the commercial pan-enterovirus q-PCR kit (DAAN Gene Co., Ltd., China) and further serotype classification was performed using commercial EV-A71, CVA-16, CVA-10, and CVA-6 q-PCR test kits with the 7500 Fast real-time PCR system (Applied Biosystems, Foster City, CA, USA) [22, 30]. All procedures were performed according to the manufacturer’s instructions. Secondly, the EV-positive samples that were not classified successfully in the previous process were amplified on the basis of the partial VP1 sequences as described earlier for further serotype identification [31]. Then, the PCR products were sequenced by Sangon Biotech (Shanghai, China). Finally, the partial VP1 sequences were analysed using The Basic Local Alignment Search Tool (www.ncbi.nlm.nih.gov/blast) and EV reference sequences available in the GenBank database.

### Phylogenetic analysis

The entire VP1 genes of CVA-6, CVA-10, and CVA-16 were amplified using specific primers: CVA-6 VP1-F: 5′- AACTTYGTRGTGCCACCAGA-3′(nucleotides 2317–2336) and CVA-6: VP1-R 5′-GTGGCGAGATGTCGGTTTA-3′ (nucleotides 3408–3426) [11]; CVA-10 VP1-F: 5′- CGRTAYTACACACARTGGTC-3′(nucleotides 2021–2040) and CVA-10 VP1-R: 5′- CTRTCYTCCCAKACHAGGTT − 3′ (nucleotides 3413–3432); CVA-16 VP1-F: 5′-AGGTACTACACCCAGTGGTCAG-3′ (nucleotides 2030–2052) and CVA-16: VP1-R 5′-GCAAGGTGCCGATTCACTACCCT-3′ (nucleotides 3400–3423) [32]. The entire procedure had been described previously [32]. The PCR products were bidirectionally sequenced by Sangon Biotech, and the sequences were submitted to Genbank under accession numbers MT119379-MT119450 and shown in Additional file. The complete sequences of the VP1 genes and those obtained from the GenBank database were aligned using the ClustalX program [33]. Phylogenetic trees were constructed using the neighbour-joining algorithm of the MEGA software 5.0 and bootstrap analysis was performed with 1000 replicates [34].

## Results

### Enterovirus seortype

**Fig. 1:**
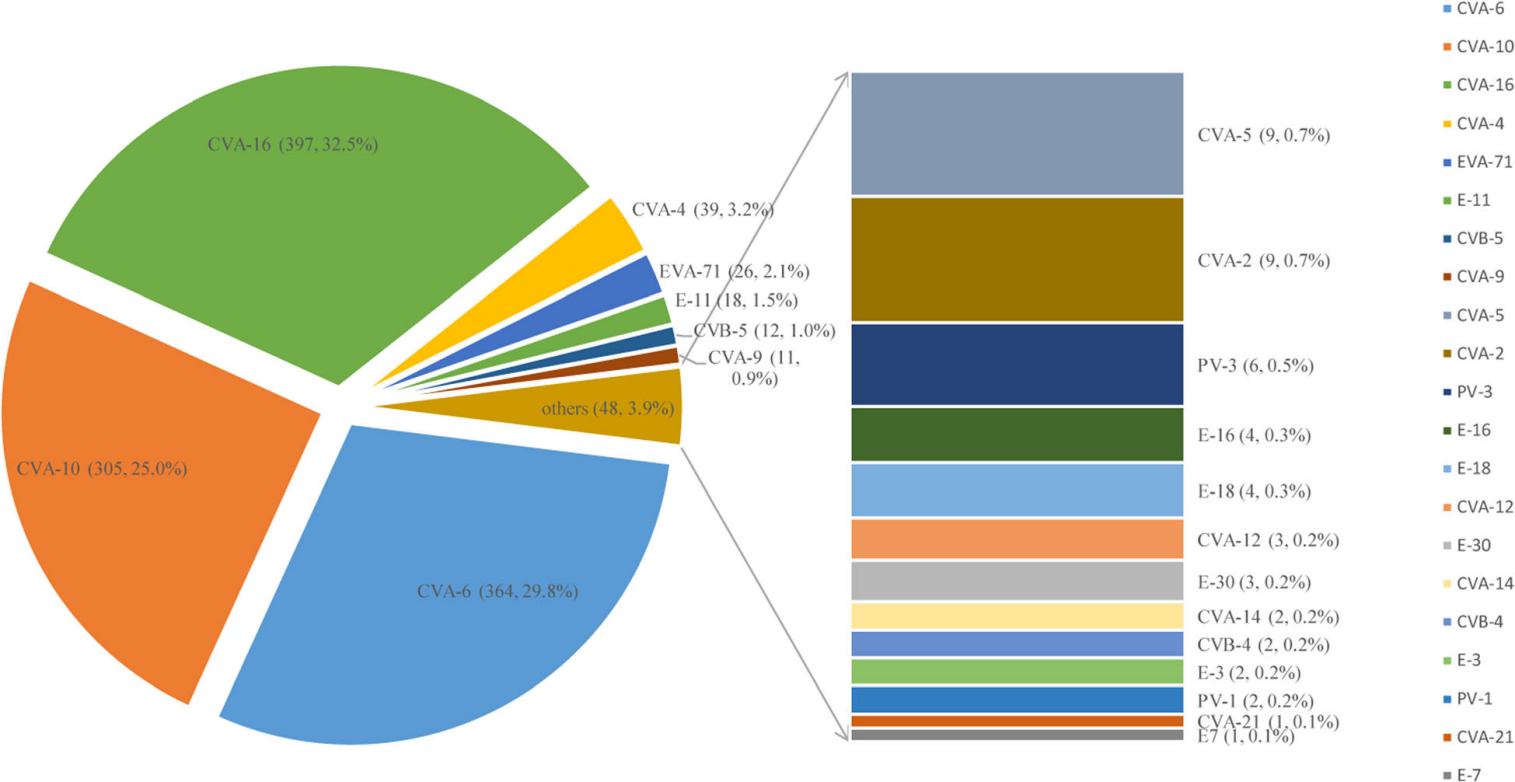
EV serotypes of HFMD patients in Guangzhou in 2018

In 2018, 1220 children with laboratory-confirmed HFMD were enrolled in this study, and the three dominant serotypes were CVA-6 (*n* = 364, 29.8%), CVA-10 (*n* = 305, 25.0%), and CVA-16 (*n* = 397, 32.5%), accounting for 87.3% of all EV-positive cases. For the remaining serotypes, the predominant serotypes were CVA-4 (*n* = 39, 3.2%), EV-A71 (*n* = 26, 2.1%), E-11 (*n* = 18, 1.5%), CVB-5 (*n* = 12, 1.0%), and CVA-9 (*n* = 11, 0.9%); some sporadic cases were closely related to CVA-5, CVA-2, PV-3, E-16, E-18, CVA-12, E-30, CVA-14, CVB-4, E-3, PV-1, CVA-21, and E-7. The detailed serotypes of EV are shown in Fig. 1.

### Diversity and distribution of EV serotypes with season

**Fig. 2:**
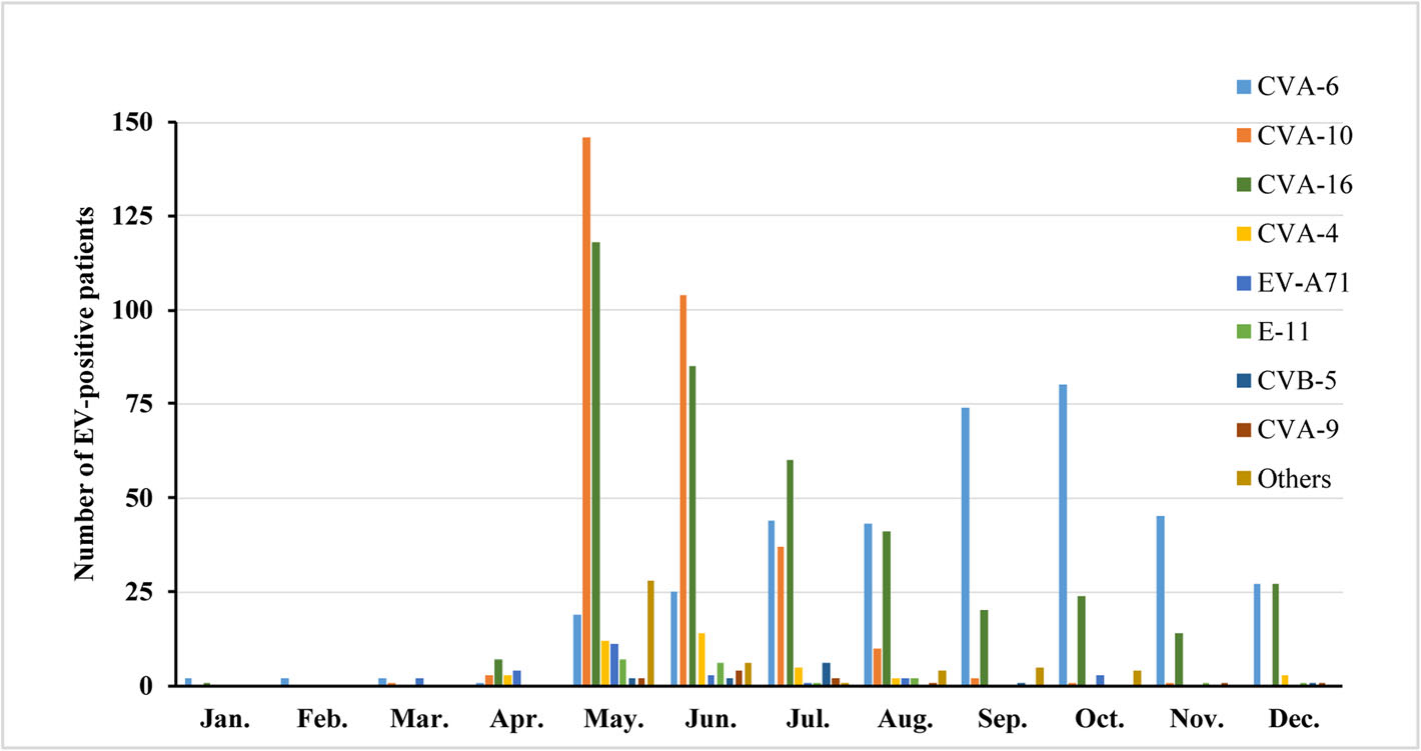
Monthly distribution of EV-positive HFMD cases

There was a significant seasonal change in the occurrences of HFMD. The majority of the patients presented from May to December in 2018. A peak in the number of cases was observed in May and June, which coincided with the co-circulation of CVA-10 and CVA-16. At the same time, there was a slight increase in the number of cases in September and October, and CVA-10 and CVA-16 were replaced by CVA-6 as the dominant EV. Thus, our data revealed a sudden increase in CVA-10- and CVA-16-positive cases in May, followed by a gradual decrease in the subsequent months. Nevertheless, the monthly prevalent pattern of CVA-6, which appeared from May and peaked in October, was considerably different from that of CVA-10 and CVA-16 (Fig. 2).

### Demographics, clinical manifestations, and laboratory data

As shown in Table 1, there was a total of 80 (6.6%) severe cases and 1140 (93.4%) mild HFMD cases were diagnosed in our study. In severe HFMD cases, the CVA-6 (*n* = 21) and CVA-10 (*n* = 18) were more frequently detected in this study, and the ratios of severe cases were 5.8 and 5.9%. The group of CVA-16 had a low ratio in severe cases compared with other four groups (*P* < 0.05). Moreover, the ratio of severe cases caused by EV-A71 was 34.6%, which was significantly higher than the other four groups (*P* < 0.001). In the others group, 26 (20.3%) cases were identified as severe HFMD, and E-11(38.9%, 7/18), CVA-4(15.4%, 6/39), and CVB-4(33.3%, 4/12) were the three main serotypes.

**Table 1:** Comparison of the severity of HFMD with different EVs infected patients

EV serotypes (N^a^)	Severe HFMD^b^	Mild HFMD^b^
CVA-6(*N* = 364)	21(5.8%)	343(94.2%)
CVA-10(*N* = 305)	18(5.9%)	287(94.1%)
CVA-16(*N* = 397)	6(1.5%)^*^	391(98.5%)
EV-A71(*N* = 26)	9(34.6%)^*^	17(65.4%)
Others(*N* = 128)	26(20.3%)^*^	102(79.7%)

^a^ The number of patients; ^b^ The number of patient (%); ^*^ Significant different compare with other groups, *P* < 0.05; Others: other EVs detected in patients except CVA-6, CVA-10, CVA-16, and EV-A71

Demographic information is shown in Table 2. There were 712 (58.4%) males and 508 (41.6%) females, with a gender ratio of 1.4:1. The majority of patients were under 5 years of age (*n* = 1152, 94.4%). The age range from 1 to 3 years was predominant among the patients, followed by 3–5 years and < 1 year, accounting for 53.8, 27.2, and 13.4% patients, respectively. Notably, 1–3-year-old children were more susceptible to EV, and there was no statistically significant difference between age distribution in different groups.

**Table 2:** Demographic data of patients affected with different EVs in this study

Demographic data					
EV serotypes	All	CVA-6	CVA-10	CVA-16	Others^a^
Total number of patients	*N* = 1220	*N* = 364	*N* = 305	*N* = 397	*N* = 154
Sex					
Male	712(58.4%)	223(18.3%)	155 (12.7%)	229 (18.8%)	105(8.6%)
Female	508(41.6%)	141(11.6%)	150(12.3%)	168 (13.8%)	49(4.0%)
Age range (years)					
< 1	164 (13.4%)	56(4.6%)	36(3.0%)	39(3.2%)	33(2.7%)
1–3	656(53.8%)	218(17.9%)	173(14.2%)	182 (14.9%)	83(6.8%)
3–5	332(27.2%)	64(5.2%)	87(7.1%)	145(11.9%)	36(3.0%)
> 5	68(5.6%)	26(2.1%)	9(0.7%)	31(2.5%)	2(0.2%)

^a^ Note: Others included all EVs detected in this study except CVA-6, CVA-10, and CVA-16

All general clinical manifestations of the 1220 HFMD patients are shown in Table 3. The data suggested that the clinical features of HFMD infected by different EV serotypes were similar. The HFMD patients commonly showed skin lesion in hands, feet, extremities and fever spike over 39 °C. The laboratory results indicated that the rising of hsCRP and WBC were commonly observed in HFMD patients. The ratio of hsCRP rising in the CVA-6 group was significantly higher than those in the other groups (*P* < 0.001), and the CVA-16 group had lower values for hsCRP than the other groups (*P* < 0.001). In addition, the CVA-10 group had higher values for both WBC and neutrophils than the other groups (*P* < 0.001).

**Table 3:** Comparison of clinical manifestations and laboratory tests with different EVs infected patients

	Total	CVA-6	CVA-10	CVA-16
Clinical symptoms and signs				
Total patients	N = 1220	N = 364	N = 305	N = 397
Cough	304(24.9%)	101(27.7%)	65(21.3%)	96(24.2%)
Diarrhea	60(4.9%)	14(3.8%)	14(4.6%)	22(5.5%)
Vomiting	228(18.7%)	60(16.5%)	67(22.0%)	79(19.9%)
Fever spike (°C)	39.3(39.0–39.8)	39.4(39.0–39.8)	39.5(39.0–39.9)	39.0(38.9–39.9)
Fever (> 38 °C)	1093(89.6%)	327(89.8%)	280(91.8%)	360(90.7%)
Neurologic complications	61(5.0%)	20(5.5%)	16(5.2%)	6(1.5%)^*^
Skin lesion	1220(100%)	364(100%)	305(100%)	397(100%)
Mouth and/or pharyngeal	1056(86.6%)	300(82.4%)	270(88.5%)	340(85.7%)
Hands and /or feet	1117(91.6%)	339(93.1%)	264(86.6%)	354(89.2%)
Trunk	210(17.2%)	76(20.9%)	51(16.7%)	72(18.1%)
Extremities	1115(91.4%)	342(94.0%)	267(87.5%)	362(91.2%)
Laboratory data				
Total patients	*N* = 1220	*N* = 364	*N* = 305	*N* = 397
hsCRP(0-10 mg/L)	6.93(2.50–17.08)	9.67(3.68–22.84)	14.26(3.92–27.3)	4.23(2.50–8.90)^*^
hsCRP (> 2.5 mg/L)	868(71.1%)	293(80.5%)^*^	236(77.4%)	246(62.0%)
WBC(5-12 × 10^9^/L)	9.9(7.8–12.6)	9.3(7.1–12.5)	11.9(8.9–14.6)^*^	9.48(7.8–11.3)
WBC > 12 × 10^9^/L	362(29.7%)	110(30.2%)	146(47.9%)^*^	74(18.6%)
Neutrophils (3-10 × 10^9^/L)	5.3(3.4–8.0)	4.3(2.7–8.1)	7.1(4.5–10.1)^*^	4.9(3.7–6.5)
Neutrophils(> 10 × 10^9^/L)	167(13.7%)	53(14.6%)	80(26.2%)^*^	20(5.0%)
Lymphocyte (1.1–3.2 × 10^9^/L)	3.1(2.3–4.1)	3.2(2.4–4.3)	3.0(2.2–3.9)	3.2(2.4–4.2)
Lymphocyte(> 3.2 × 10^9^/L)	563(46.1%)	197(49.2%)	126(41.3%)	196(49.4%)
PLT (150-400 × 10^9^/L)	274(231–329)	274(232–334)	273(229–339)	278(227–324)
PLT(> 400 × 10^9^/L)	109(8.9%)	30(8.2%)	26(8.5%)	36(9.1%)
Total patients	*N* = 758	*N* = 236	*N* = 204	*N* = 246
AST (30-40 U/L)	36(31–41)	35(30–41)	36(32–40)	36(31–41)
AST(> 40 U/L)	183(24.1%)	60(25.4%)	45(22.1%)	66(26.8%)
CK (30-135 U/L)	94(72–118)	90(69–111)	96(75–120)	97(72–126)
CK(> 135 U/L)	126(16.6%)	50(21.2%)	36(17.6%)	34(13.8%)
CK-MB(0-25 U/L)	20(16–25)	21(16–25)	19(15–24)	22(16–25)
CK-MB(> 25 U/L)	169(22.3%)	52(22.0%)	43(21.1%)	60(24.4%)

Continuous data are presented as median (interquartile range) and categorical data are presented as n (%)

* Significant different compare with other groups, *P* < 0.05

### Phylogenetic analysis

In order to better understand the molecular epidemiology of HFMD in Guangzhou, the predominant serotypes, CVA-6, CVA-10, and CVA-16, were selected to phylogenetic analysis. To analyse the CVA-6, CVA-10, and CVA-16 genotypes in the specimens, we amplified the VP1 gene of the viruses. In total, 72 complete VP1 genes from CVA-6 (*n* = 24), CVA-10 (*n* = 24), and CVA-16 (*n* = 24) were amplified and sequenced. Then, the complete VP1 nucleotide sequences of CVA-6, CVA-10, and CVA-16 were determined (915 bp, 894 bp, and 891 bp, respectively), and reference sequences from elsewhere in China (*n* = 150, 111, and 130, respectively) and other countries (*n* = 8, 8, and 33, respectively) elected from the GenBank were used for phylogenetic analyses.

**Fig. 3:**
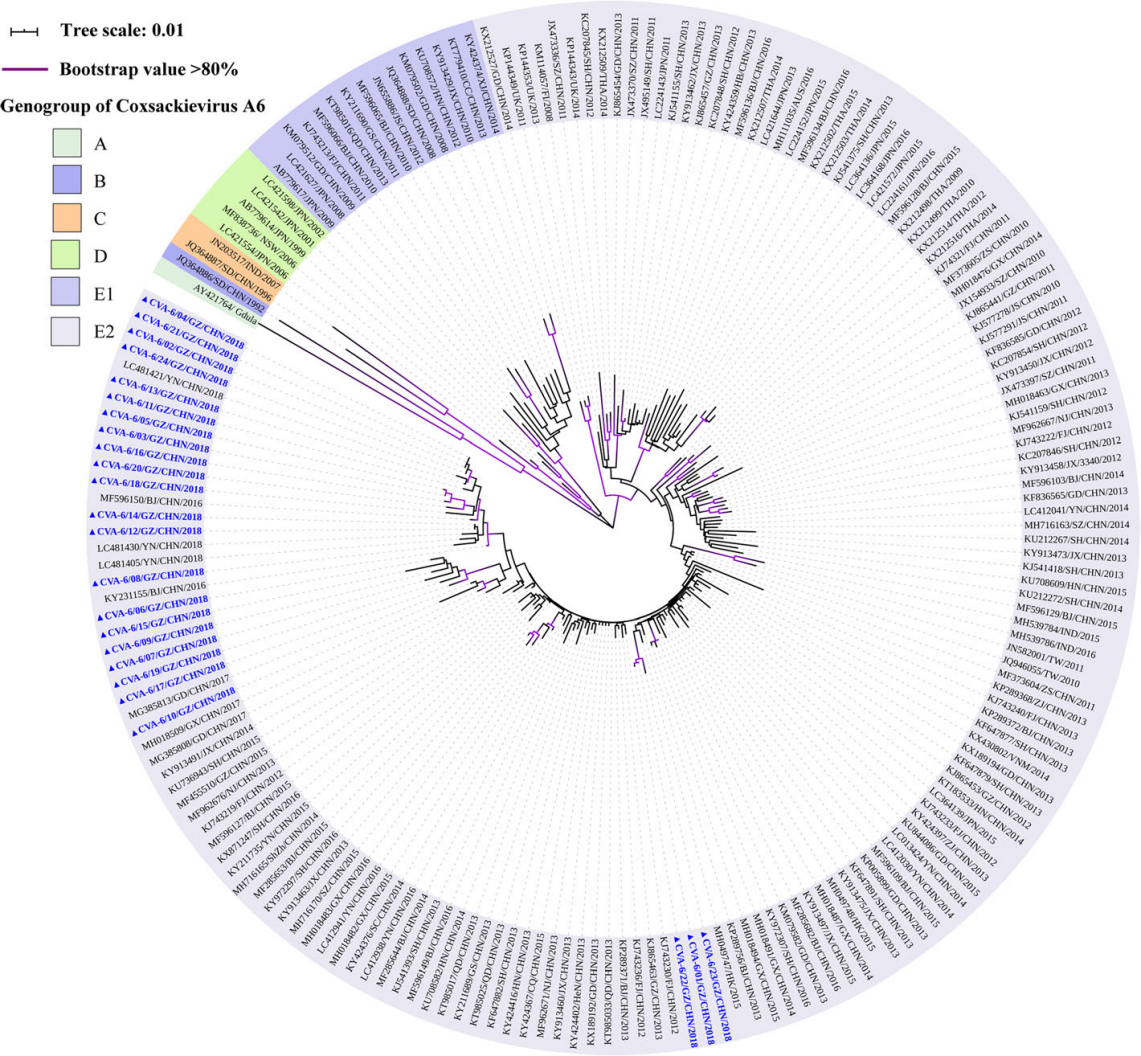
Neighbour-joining tree of the complete VP1 gene (915 nucleotides) of the CVA-6. The CVA-6 sequences (CVA-6/01/GZ/CHN/2018- CVA-6/24/GZ/CHN/2018) obtained from patients with HFMD in this study are marked by a triangle, and 158 reference sequences from elsewhere elected from the GenBank were used for phylogenetic analyses. The reference sequences were presented as GenBank accession number/province/country/year of isolation. The genogroup of CVA-6 has been divided into A, B, C, D, E1, and E2. The scale bars indicate the number of nucleotide substitutions per site. Bootstrap values were calculated on 1000 replicates. Phylogenetic nodes with bootstrap values over 80% are marked with purple lines

The CVA-6  2018 Guangzhou sequences shared 94.4–99.9% and 98.4–100% of nucleotide and amino acid identity level respectively as well as 82.4–84.3% and 95.4–96.4% nucleotide and amino acid identity respect to the CVA-6 prototype (CVA-6/Gdula, GenBank accession no. AY421764) respectively. The phylogenetic tree indicated that all 24 CVA-6 belonged to sub-genogroup E2, and are closely related to strains found in Hong Kong, Yunnan, Guangdong, and Guangxi during 2015–2018 (Fig. 3).

**Fig. 4:**
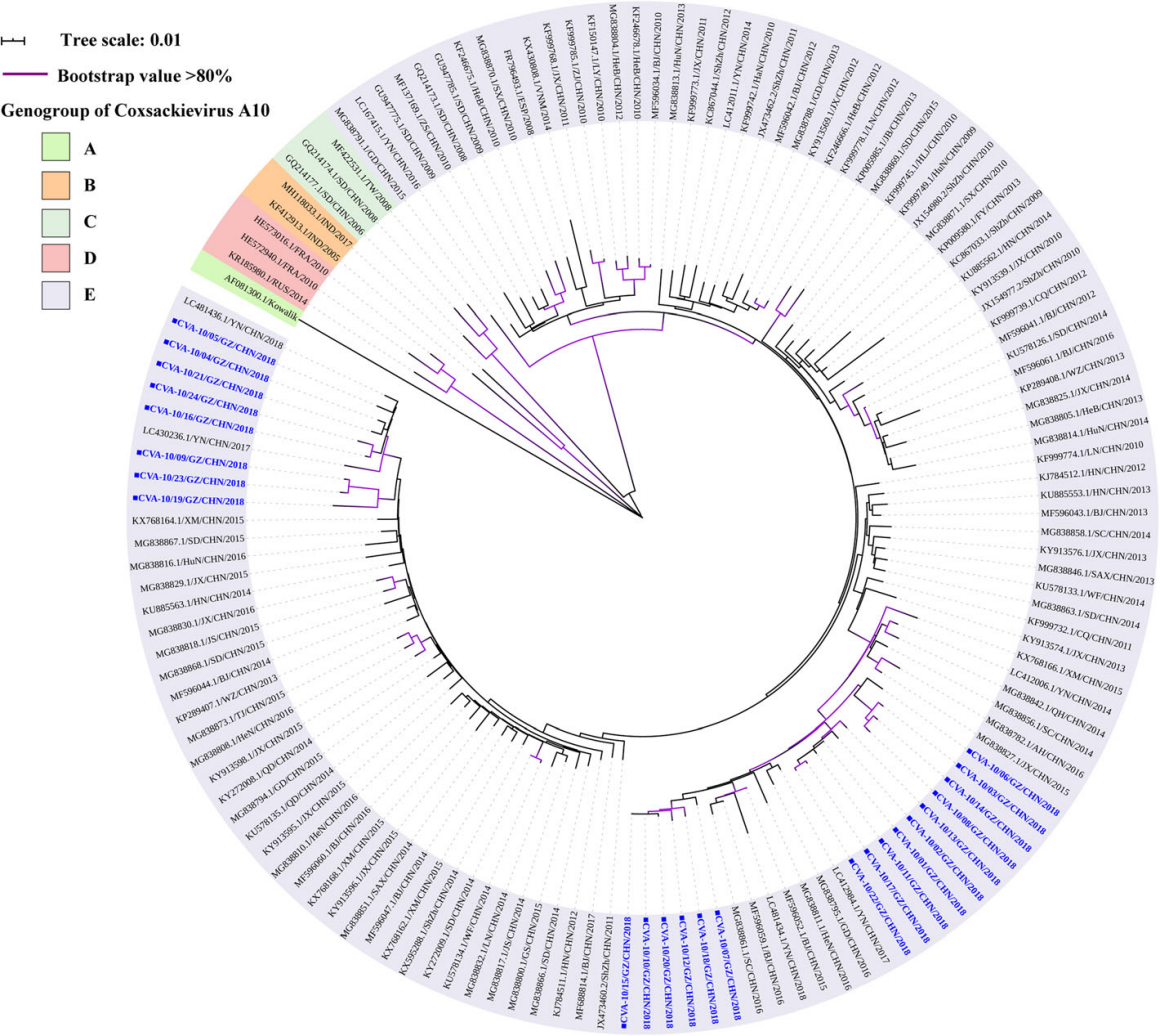
Neighbour-joining tree of the complete VP1 gene (894 nucleotides) of the CVA-10. The CVA-10 sequences (CVA-10/01/GZ/CHN/2018- CVA-10/24/GZ/CHN/2018) obtained from patients with HFMD in this study are marked by a diamond, and 119 reference sequences from elsewhere elected from the GenBank are used for phylogenetic analyses. The reference sequences are presented as GenBank accession number/province/country/year of isolation. The genogroup of CVA-10 has been divided into A, B, C, D, and E. The scale bars indicate the number of nucleotide substitutions per site. Bootstrap values were calculated on 1000 replicates. Phylogenetic nodes with bootstrap values over 80% are marked with purple lines

The CVA-10 2018 Guangzhou sequences shared 92.2–99.9% and 97.3–100% of nucleotide and amino acid identity level respectively as well as 75.6–77.1% and 92.0–93.6% nucleotide and amino acid identity respect to the CVA-10 prototype (CVA-10/Kowalik, GenBank accession no. AF081300) respectively. All CVA-10 in our study belonged to the genogroup E. CVA-10 Guangzhou showed high homology with strains previously isolated from the Yunnan, Guangdong, Henan, and Sichuan provinces (Fig. 4).

**Fig. 5:**
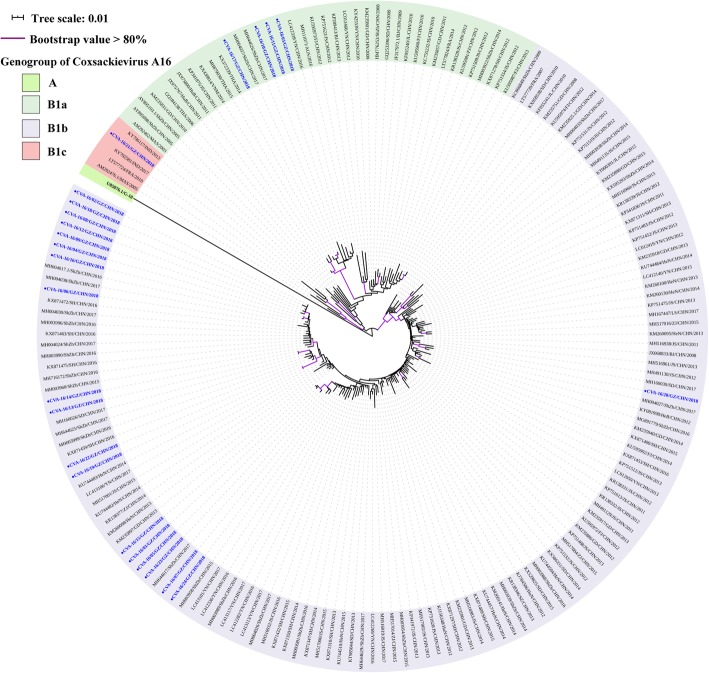
Neighbour-joining tree of the complete VP1 gene (891 nucleotides) of the CVA-16. The CVA-16 sequences (CVA-16/01/GZ/CHN/2018- CVA-16/24/GZ/CHN/2018) obtained from patients with HFMD in this study are marked by a circle, and 163 reference sequences from elsewhere elected from the GenBank are used for phylogenetic analyses. The reference sequences are presented as GenBank accession number/province/country/year of isolation. The genogroup of CVA-16 has been divided into A, B1a, B1b, and B1c. The scale bars indicate the number of nucleotide substitutions per site. Bootstrap values were calculated on 1000 replicates. Phylogenetic nodes with bootstrap values over 80% are marked with purple lines

The CVA-16 2018 Guangzhou sequences shared 87.3 to 99.9% and 98.0 to 100% of nucleotide and amino acid identity level respectively as well as 74.8 to 76.2% and 91.3 to 92.3% nucleotide and amino acid identity respect to the CVA-16 prototype (CVA-16/G10, GenBank accession no. U05876) respectively. The CVA-16 phylogenetic dendrogram revealed that all Guangzhou CVA-16 were grouped into genogroup B. Twenty-three sequences belonged to sub-genogroup B1a and B1b, which share a close genetic evolutionary relationship with subtypes that circulated in Yunnan, Thailand, Shenzhen, Shandong, and Shanghai from 2014 to 2017. The remaining one CVA-16 sequence belonged to sub-genogroup B1c and clustered with isolates from India identified in 2017 (GenBank accession no. KY792581). It was also the first case to be reported in China (Fig. 5).

## Discussion

Currently, EV-A71 and CVA-16 are still the main known causative agents of HFMD. However, CVA-6 and CVA-10 were frequently detected in HFMD outbreaks in recent years [19, 32, 35], which implied that the epidemiological features of HFMD are changing. In our study, we reported for the first time a new HFMD epidemic pattern in Guangzhou in 2018 characterised by co-circulation of CVA-6, CVA-10, and CVA-16. Additionally, seasonally circulating diversity in serotypes was observed in this study, which provided a scientific basis for prevention and control of HFMD in the future.

In this study, our results showed that the co-circulation of three main EV serotypes (CVA-6, CVA-10, and CVA-16) caused the HFMD outbreak in Guangzhou, which was clearly different with the epidemic characteristics described before. In previous study, EV-A71 and CVA-16 had been reported to the predominant pathogens of HFMD in Guangzhou between 2008 and 2010 [26, 36]. However, the number of CVA-6 has been increased dramatically since 2010, and CVA-6 has become the major pathogen in 2017 [22, 25]. In 2018, CVA-6, CVA-10, and CVA-16 had been co-circulating in the HFMD outbreak in Guangzhou. Based on the limited co-circulating information, we suspected it was associated with genetic variation [19, 37], and further studies were needed to verified it. According to previous studies, EV-A71 was frequently detected in China and considered to be closely associated with fatal case due to HFMD outbreak worldwide [4, 36, 38, 39]. Our results indicated that 26 EV-A71-positive HFMD cases were identified, and the ratio of HFMD severe was significantly higher in the group of EV-A71. The relatively lower viral diversity of EV-A71 and successful application of EV-A71 vaccine may be responsible for the reduced incidence of EV-A71 [40–42]. Moreover, we still need to keep our eyes on the HFMD caused by EV-A71 even the number of it was low. Up to date, only CVA-16 and EV-A71 were identified as the main pathogens in the Chinese nationwide surveillance of HFMD. The molecular epidemiological data of this study demonstrated that multi-serotypes circulated concomitantly, which might become a HFMD epidemic trend in the future. Notably, several polioviruses (PV) were detected in this study. PV type 1–3, belonging to EV-C, is notable as the pathogen of poliomyelitis (polio) [17, 43]. We observed that all the patients associated with PV presented typical clinical manifestation of HFMD, such as skin rashes on hands and feet, and the age with serotypes of the affected associated with the PV inoculation [44, 45]. However, the vaccination records of eight patients were unclear and further studies are needed. In addition, our data indicated that apart from CVA-6, CVA-10, and CVA-16, a total of 18 enterovirus serotypes were associated with HFMD cases, which had been described in previous studies [32, 46]. However, most of these serotypes have been rarely detected in clinical laboratories. Therefore, the development of a detection kit which is capable of detecting more enterovirus serotypes is crucial for clinical diagnosis of HFMD. Further, it is vital for researchers and the Centre for Disease Control and Prevention to pay attention to other EVs [47, 48]. This is especially important as only the commercial EV-A71 vaccine, which lacks cross immunity protection for patients infected with other EVs, has been successfully applied to children [47]. Our result implicated that the development of multi-serotype vaccines will be helpful for decreasing the morbidity associated with HFMD.

A seasonal pattern of HFMD was observed in this study. There were two peaks when the majority of cases were admitted to the hospital, and one major peak in May and a small peak in October. This phenomenon was consistent with some earlier studies reported in Guangzhou and other southern regions, including Thailand and Taiwan [26, 38, 49–51]. The exact reason was still unclear. But climate and geographical location were thought to be involved in the seasonal change of HFMD to a certain extent. High temperature and high humidity had increased the incidence of HFMD, and it had also been found that atmospheric pressure and wind speed were associated with HFMD occurrence [51]. Additionally, different serotypes of EV were distinct in their monthly distribution. Significantly, CVA-6 was the predominant pathogen of the small HFMD peak in autumn, and CVA-10 and CVA-16 were prevalent in the beginning of summer (the peak in May), which was similar to that observed in Shanghai and southern Vietnam to a certain extent [10, 52]. The discrepancy in the monthly distribution of CVA-6 and CVA-10 in different regions may be attributed to the adaptive changes in the pathogens. Hence, it is crucial to develop reasonable preventive measures to combat the HFMD outbreak caused by various serotypes in different seasons.

In this study, EVs have an obvious predilection in patients. The majority of patients were under 5 years of age in this study, which is possibly associated with the incomplete immunity of children [3]. The incidence rate in boys with HFMD was higher than that of girls, with 1.4:1 sex ratio, which was similar to the results of previous studies [10, 49, 52]. We suspected that poor hygiene, more outdoor activities, and host factors may be related to that phenomenon [22]. Moreover, our results did not show any age or gender predilection in different serotypes. Compared to the group of CVA-10 and CVA-16, patients infected with CVA-6 were likely to have the rising hsCRP. Moreover, the group of CVA-10 had an obvious increase in WBC, which was caused by the rising of neutrophils. However, further studies regarding this are required as the existing information is limited.

Based on the entire VP1 sequence, low intra-typic divergence was observed among CVA-6, CVA-10, and CVA-16 detected from Guangzhou. The first reported Chinese CVA-6 strain was grouped to genogroup B. Subsequently, the sub-genogroup E2, which replaced genogroups B, C, D, and E1, has gradually become the predominant strain circulating worldwide since 2008 [12]. The CVA-6 Guangzhou were further segregated into two groups within sub-genotype E2. One cluster was close to the subtypes detected in Yunnan, Guangdong, and Guangxi, while the other was closely related to strains from Hong Kong and Fujian. Both CVA-6 clusters were similar to the strains in adjacent provinces of Guangzhou, which indicated that the subtypes of CVA-6 identified in Guangzhou had a common ancestor in nearby regions and are prevalent in the south of China. Of the CVA-10, Guangzhou sequences were identified to genogroup E, which was prevalent not only in Guangzhou, but also in Xiamen, Yunnan, and Jiangxi. The evolutionary characteristics of sub-genotype E are still not known and further studies are required to elucidate the increase in CVA-10 prevalence in Guangzhou during 2018. The CVA-16 phylogenetic dendrogram showed that genogroup B, consisting of the majority of CVA-16, was classified into B1a, B1b, and B1c. Furthermore, sub-genogroups B1a and B1b associated with outbreaks of HFMD in Nanjing, Fujian, and Yantai, were recognised as the predominant subtypes circulating around the mainland [39, 53–55], which was consistent with the results of the present study in Guangzhou. The CVA-16 phylogenetic dendrogram indicated that sub-genogroup B1b, including 19 Guangzhou CVA-16, was dominant in our study, similar to the results of previous reports on other regions [53, 55]. The Guangzhou CVA-16 located in the B1b group showed high similarity to the strain isolated in Shenzhen, a city adjacent to the Guangdong province. Additionally, four CVA-16 sequences belonging to the B1a sub-genogroup were closely related to strains circulating in Shenzhen and Yunnan. Therefore, we hypothesised that compared to B1a, the sub-genogroup B1b shares a common ancestor with strains circulating in Shenzhen, which might become endemic strains of CVA-16 in the future. Intriguingly, one sequence was located in the sub-genogroup B1c, which has been reported only in Malaysia, France, and India [16, 54]. Hence, we believed that one located in B1c was obtained from other countries via import transmission [55]. Comprehensive and long-term surveillance of HFMD is critical for understanding the trend of the epidemic and emergence of new genotypes, and for controlling EV infection in the future.

Our study has several limitations. For example, this study was not a multi-center and multi-year research, and some laboratory diagnosis lacked in this study, which may cause a bias to the different serotypes in laboratory data.

## Conclusions

We found that a total of 21 EV serotypes were identified in 1220 HFMD patients, and CVA-6, CVA-10, and CVA-16 were the most commonly detected during 2018. Co-circulation of three serotypes, CVA-6, CVA-10, and CVA-16, was the dominant reason for the outbreak of HFMD in Guangzhou in 2018. In this study, we presented a comprehensive and detailed investigation regarding the outbreak of HFMD in Guangzhou based on clinical manifestations and phylogenetic analysis, which we believe will be beneficial for understanding and controlling the disease in the near future.

## Supplementary information

**Additional file 1.** Fasta-formatted file of the 72 VP1 gene sequences used in phylogenetic analysis.

## Data Availability

The datasets generated and/or analysed during the current study are available in the GenBank repository under accession numbers MT119379-MT119450. The datasets generated and/or analysed during the current study are also available in Additional files.

## Abbreviations

HFMD: Hand, foot, and mouth disease
EV-A: Enterovirus A
CVA: Coxsackievirus A
CVB: Coxsackievirus B
EV: Enterovirus
VP: Viral protein
E: Echovirus
q-PCR: Quantitative real-time PCR
RT-PCR: Reverse transcription reverse PCR
hsCRP: Hypersensitive C-reactive protein
WBC: White blood cell
PLT: Platelet
AST: Aspartate aminotransferase
CK: Creatine kinase
CK-MB: Creatine kinase-MB
PV: Poliovirus
